# The long non-coding RNA HOXA11-AS activates ITGB3 expression to promote the migration and invasion of gastric cancer by sponging miR-124-3p

**DOI:** 10.1186/s12935-021-02255-6

**Published:** 2021-10-29

**Authors:** Liting You, Qian Wu, Zhaodan Xin, Huiyu Zhong, Juan Zhou, Lin Jiao, Xingbo Song, Binwu Ying

**Affiliations:** 1 Department of Laboratory Medicine, West China Hospital, Sichuan University, 37, Guoxue Lane, Chengdu, 610041 Sichuan China; 2 Department of Clinical Laboratory, Renmin Hospital of Wuhan University, Wuhan, 430060 Hubei China

**Keywords:** HOXA11-AS, Gastric cancer, ITGB3, miR-124-3p

## Abstract

**Background:**

miR-124-3p can inhibit integrin β3 (ITGB3) expression to suppress the migration and invasion of gastric cancer (GC), and in the process lncRNA HOXA11-AS may act as a molecular sponge.

**Methods:**

Luciferase reporter assay was conducted to verify the binding of miR-124-3p and HOXA11-AS. RT-PCR and western blot were performed to detect the expression of HOXA11-AS, miR-124-3p and ITGB3 in GC tissues and cells. Gene silence and overexpression experiments as well as cell migration and invasion assays on GC cell lines were performed to determine the regulation of molecular pathways, HOXA11-AS/miR-124-3p/ITGB3. Furthermore, the role of HOXA11-AS in GC was confirmed in mice models.

**Results:**

We found HOXA11-AS is up-regulated in GC tissues and can bind with miR-124-3p. Through overexpression/knockdown experiments and function tests in vitro, we demonstrated HOXA11-AS can promote ITGB3 expression by sponging miR-124-3p, consequently enhance the proliferation, migration, and invasion of GC cells. Meanwhile, we validated that HOXA11-AS promotes migration and invasion of GC cells via down-regulating miR-124-3p and up-regulating ITGB3 in vivo.

**Conclusions:**

We demonstrated that lncRNA HOXA11-AS can increase ITGB3 expression to promote the migration and invasion of gastric cancer by sponging miR-124-3p. Our results suggested that HOXA11-AS may reasonably serve as a promising diagnostic biomarker and a potential therapeutic target of GC.

**Supplementary Information:**

The online version contains supplementary material available at 10.1186/s12935-021-02255-6.

## Background

Gastric cancer (GC) is one of the most aggressive digestive cancers and the leading causes of cancer-related deaths worldwide [1, 2]. Despite the fast advancements of diagnostic and therapeutic strategies in recent years, the 5-year survival rate of GC patients remains unsatisfactory mainly owing to early progression and metastasis. Malignancies are well-known molecular diseases caused by the variation accumulation of tumor-associated genes. In the past decades, plenty of molecules associated with progression and metastasis of GC have been discovered; yet, the mechanisms underlying GC invasiveness remain poorly understood.

Integrin β3 (ITGB3) can promote GC invasion and metastasis and some micro-RNAs (miRNAs) can regulate the expression of ITGB3. Previous studies demonstrated that miRNA-124-3p (miR-124-3p) can down-regulate ITGB3 expression and inhibit gastric cancer cell proliferation and migration [3–6]. It has been confirmed that miR-124-3p can decrease ITGB3 mRNA by binding to its 3′ untranslated region (3′ UTR), and in reverse, inhibiting miR-124-3p can promote ITGB3 expression. Our previous study also explored the action of miR-124-3p/ITGB3 in GC invasion and migration [7]. We revealed miR-124-3p can bound to partial complementary sites of ITGB3 mRNA and induce post-transcriptional silencing of the ITGB3 gene to inhibit gastric cancer cell proliferation and invasion. However, the molecular mechanisms underlying the role of miR-124-3p/ITGB3 in GC progression are still not well documented.

LncRNA HOXA11-AS is a long non-coding RNA encoded by HOXA11 antisense gene which is located on chromosome 7p15.2 [8]. It plays an important role in the occurrence and progression of multiple disease. For example, lncRNA HOXA11-AS functions as an oncogene in malignancies including glioma, hepatocellular carcinoma (HCC), non-small cell lung cancer (NSCLC), osteosarcoma, gastric cancer (GC) and et al. [9]. Competing endogenous RNAs (ceRNAs) are one of the most common mechanisms of long non-coding RNAs (lncRNAs) function. LncRNAs can increase mRNA transcripts by competitively binding to miRNA response element (MRE) [10]. LncRNA HOXA11-AS participates in tumor progression and metastasis through regulating the expression of numerous pathways and genes [11, 12]. Preliminary studies have shown that lncRNA HOXA11-AS was present in GC and can promote GC cells proliferation, migration and invasion. And it was demonstrated in osteosarcoma that lncRNA HOXA11-AS can up-regulate ROCK1 gene expression by sponging miR-124-3p [8]. However, in GC, the functions of HOXA11-AS and its association with miR-124-3p/ITGB3 remain largely unknown.

In this study, we identified lncRNA HOXA11-AS as ITGB3 mRNA’s competitor in binding with miR-124-3p MRE sites. HOXA11-AS expression in GC tissues was higher than that in paired adjacent normal tissues. By overexpression of HOXA11-AS both in vitro and in vivo, we detected miR-124-3p down-regulation and ITGB3 up-regulation and observed significant enhancements of GC invasiveness, and in reverse by silence of HOXA11-AS, the opposite effects were observed. Our data showed HOXA11-AS can bind with miR-124-3p and function as a molecular sponge for miR-124-3p to increase the expression of ITGB3. Furthermore, HOXA11-AS promotes GC migration and invasion via regulating miR-124-3p/ITGB3.

## Methods

### Patients and tissue samples

As we have described in our previous study [7], 40 pairs of GC patients’ tissues and adjacent normal tissues were collected from March to September 2017 in West China Hospital, Sichuan University. Cancer and adjacent normal tissues of patients with definite pathological diagnosis of gastric cancer were included in our study, regardless of tumor size, clinical stage and pathological grades. These patients did not undergo any adjuvant therapy before surgery. Part of tissues were rapidly frozen in liquid nitrogen immediately after removal, and the other tissues were fixed in 4% paraformaldehyde and DEPC-treated paraformaldehyde, respectively.

### Cell culture and transfection

MKN-45, MGC803, AGS human gastric cancer cell lines and HEK-293T cell line were purchased from Procell Life Science&Technology Co., Ltd (Wuhan, China) in Jan 2019. All cell lines underwent short tandem repeats (STR)-authentication and mycoplasma contamination tests. And all cells were cultured with Dulbecco’s Modified Eagle Medium (DMEM) (Invitrogen, Carlsbad, CA, USA) containing 10% fetal bovine serum (FBS) (Hyclone, Logan City, UT, USA) and 1% penicillin/streptomycin (Hyclone, Logan City, UT, USA) in a humidified atmosphere of 5% CO2 at 37 °C. We performed rapid freezing cryopreservation for first two to four generations of cells with 1mL freezing medium of 90% FBS and 10% Dimethyl sulfoxide (DMSO) in liquid nitrogen.

SiRNA against lncRNA HOXA11-AS (si-HOXA11-AS), negative control siRNA of random sequence (siRNA-NC), miR-124-3p mimics, negative control miR-124-3p mimics (mimics NC), miR-124-3p inhibitor (ASO-miR-124-3p), and negative control miR-124-3p inhibitor (ASO-NC) were all purchased from Genechem Co., Ltd, (Shanghai, China). HOXA11-AS cDNA sequences were amplified and cloned to pcDNA3.1 carrier (Invitrogen, Carlsbad, CA, USA). According to the manufacturer’s instructions of Lipofectamine 2000 (Invitrogen, Carlsbad, CA, USA), cells transfection was conducted in six-well plates.

### Luciferase reporter assay

Luciferase reporter assay was conducted to verify the binding association of lncRNA HOXA11-AS and miR-124-3p. PsiCEHCK-2 dual luciferase vectors (Promega Corporation, Fitchburg, WI, USA) respectively with the wild type of HOXA11-AS (WT-HOXA11-AS) and the mutant type of HOXA11-AS (MUT-HOXA11-AS) were constructed. The primer sequences of the wild-type and mutant HOXA11-AS are listed in Additional file 1: Table S1. Cells transiently co-transfections with luciferase reporter plasmid were conducted in HEK-293 T cells in following groups: PsiCEHCK-2 with wild-type (WT)-HOXA11-AS and miR-124-3p mimics, PsiCEHCK-2 with WT-HOXA11-AS and miR-124-3p mimics-NC, MUT (mutant)-HOXA11-AS and miR-124-3p mimics, or MUT-HOXA11-AS and miR-124-3p mimics-NC. After 48 h of incubation at 37 °C, we collected the transfected cells to measure the luciferase activity value with the Dual-Luciferase Reporter Assay System (Promega, Fitchburg, WI, USA).

### RNA extraction and real-time PCR (RT-PCR)

The total RNAs of patient’s tissues or cultured cells were extracted according to our previous protocol [7]. Then we used the PrimeScript ™ RT reagent Kit with gDNA Eraser (TaKaRa, Japan) to remove genomic DNA and conduct cDNA synthesis reaction. Real-time PCR was performed using SYBR® PrimeScript™ miRNA RT-PCR Kit (Takara, Japan) according to the manufacturer’s instruction. U6 and GAPDH were used as endogenous controls. The relative expression levels were calculated by 2^−ΔCt^ method. The primer sequences used are listed in Additional file 1: Table S2.

### GEPIA and Lnc2Cancer 3.0 database

The online Gene Expression Profiling Interactive Analysis (GEPIA) (http://gepia.cancer-pku.cn/) [13] database was used to compare the expression level of lncRNA HOXA11-AS in STAD tissues with that in normal tissues. All STAD (n = 408) of any stages and grades as well as matched TCGA normal and GTEx data (n = 211) were selected, detailed information of clinical stage was not showed in GEPIA database. Differential analysis was performed by using disease state (Tumor or Normal) as a variable, and statistical method was one-way ANOVA. The log2 [TPM (transcripts per kilobase million) + 1] was log scale and the Jitter size was 0.4. The P-value cutoff was 0.01. Lnc2Cancer 3.0 was also used to compare the expression level of lncRNA HOXA11-AS in STAD tissues with that in normal tissues by using its RNA-seq Web Tools and to conduct survival analysis based on HOXA11-AS expression (http://www.bio-bigdata.com/lnc2cancer/) [14]. Differential analysis was performed by using disease state (Tumor or Normal) as a variable, and statistical method was Kruskal-Wallis test. The log2 [TPM (transcripts per kilobase million) + 1] was log scale. Kaplan–Meier survival analysis was performed based on median lncRNA HOXA11-AS expression. Statistical significance was assessed by using the log-rank test.

### Gene Expression Omnibus (GEO) database

We searched array sequencing data on the Gene Expression Omnibus (GEO) database for the following keywords: “Gastric cancer” and “Homo sapiens” [15]. And then we selected the study type as “Expression profiling by array” and deleted the datasets over 10 years ago. Through further screening, we removed the datasets with undetected or extremely low expression of HOXA11-AS, datasets without normal gastric controls, as well as datasets that only provided expression profiling of human GC cell lines. After that, we obtained the three datasets: GSE103236, GSE158662 and GSE79973. By using online tools GEO2R, we performed differentially expression analyses for gastric cancer versus normal tissues. Log 2 (Fold Change) and *P* value were recorded in the Additional file 1: Table S3.

### K–M plotter

The correlation between HOXA11-AS expression and survival of GC patients was analyzed by using the Kaplan–Meier plotter (http://kmplot.com/analysis/). We selected gastric cancer datasets and searched 239950_at (HOXA11-AS) to analyze both overall survival (OS) and first progress survival (FPS) [16]. The hazard ratio (HR) with a 95% confidence interval (CI) and log-rank *P* value were computed. We selected the best expression cutoff to distinguish survival.

### Western blotting

According to our previous protocol [7], the proteins were extracted and separated by SDS-PAGE and then transferred to PVDF membranes (Millipore, Billerica, MA, USA). And the membranes were separately incubated with anti-ITGB3 antibody (Abcam, USA), anti-GAPDH or anti-ACTIN antibody (Abcam, USA), or HRP-labeled goat anti-rabbit IgG secondary antibody (Abcam, USA). Pictures of the film strips were taken by using the gel imaging and analysis system (LabWorksTM) (UVP, USA) and the brightness values of the strips were analyzed.

### Wound-healing assay

To assess the migration ability of cells, wound healing assays were conducted. We seeded cells of each group in 6-well plates at an initial density of 5 × 10^5^ cells per well. And then, we used micropipette tip to make a scratch across the center of the well when cells reached 90% confluence. Then the wells were washed twice with serum-free ECM medium (Invitrogen, Carlsbad, CA, USA) and added with ECM complete medium after that. Photographs were taken at 0 and 24 h (Olympus BX53 microscope), respectively. The width of every scratch was measured using Image-Pro Plus 6.0.

### Transwell migration and invasion assays

According to our previous protocols for transwell migration assay, we seeded 2 × 10^5^ MGC-803 cells or 5 × 10^5^ MKN45 cells into the upper chamber of transwell chambers, and that, the lower chambers were supplied with 20% Gibco-contained medium. And the remaining cells in the chambers were removed and washed off after 72 h incubation. Migratory cells were immobilized with methanol; dyed in crystal violet staining solution; taken pictures (Olympus BX53 microscope); and counted. The transwell invasion assay was the same as the transwell migration assay except the step that adding 50 µL/well dissolving matrigel (BD Biosciences, Bedford, MA) medium in the upper chamber.

### CCK-8 assay

Cell proliferation capacity was evaluated by using Cell Counting Kit-8 (CCK-8) (Dojindo, Japan). The transfected cells were seeded in 96-well plates with 3000 cells/well respectively. At the time point of 12 h, 24 h, 48 h, 72 h after incubation, add 10 µL of CCK8 solution into each well and determine the cell viability. Absorbance values were measured using MRX II microplate reader (Dynex Technologies, Chantilly, VA, USA) at 450 nm.

### Gastric cancer xenograft animal models

Female BALB/c-nu nude mice (n = 24) were purchased from the Institute of Medical Laboratory Animals, Chinese Academy of Medical Sciences. Six random mice were used for each group. The selected stable AGS/HOXA11-AS, AGS/Control, AGS/sh-HOXA11-AS, AGS/sh-NC cells were cultured separately, and expanded to about 80–90% density. 1 × 10^6^ cells of these four groups were subcutaneously injected into the mice of each group, respectively. The weight of mice was assessed every 7 days, and the length of the tumor was measured every 3 days. After 3–5 weeks, the nude mice were anesthetized and photographed to observe the difference in tumor formation of the four groups. Then the tumors were completely peeled off, and the tumor tissues were weighed and photographed. The tumor specimens were divided into two parts subsequently, one part was fresh tumor tissue stored at − 80 °C, and the other was fixed with neutral formalin. The expression level of HOXA11-AS, miR-124-3p and ITGB3 in tumor tissues was detected by RT-PCR. The expression of Integrin β3, Ki-67 and CD34 was analyzed by immunohistochemical (IHC) staining.

### H&E and IHC staining

Mice tumor tissues of four groups were fixed in 4% paraformaldehyde and were performed dehydration, transparency, wax immersion, embedding, poly-lysine coating on the glass slide. After dewaxing, the slides were stained with hematoxylin and eosin, and were photographed with the Olympus BX53 microscope. Parts of the tumor tissues were fixed in paraformaldehyde, and the expressions of integrin β3, Ki67, and CD34 in each group of tissues were detected by IHC analyses.

### Statistical analysis

Independent sample t-test or paired samples t-test was used for the comparison between two groups under different conditions. ANOVA analysis of variance was used for comparison between multiple groups. All tests are two-sided probabilities, and the differences with *P* value <0.05 were considered statistically significant. Above statistics analyses were performed by SPSS 22.0 (Chicago, IL, USA) and GraphPad Prism 6.0 (GraphPad Software, La Jolla, CA, USA).

## Results

### The lncRNA HOXA11-AS is up-regulated in GC tissues and correlated with miR-124-3p and ITGB3 expression

**Fig. 1:**
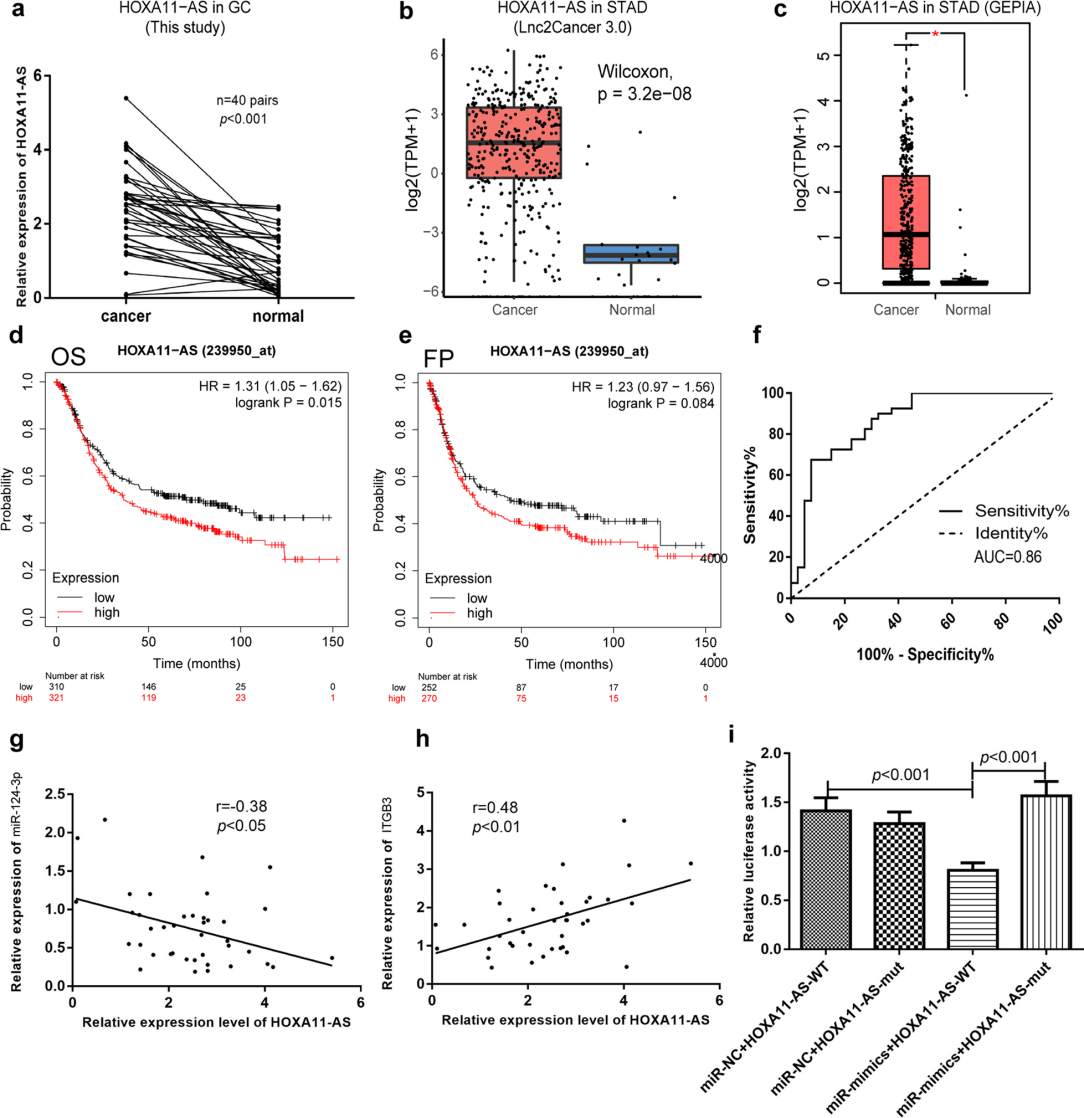
The lncRNA HOXA11-AS is up-regulated and correlated with miR-124-3p and ITGB3 RNA levels in gastric cancer tissues of patients. Expression of HOXA11-AS in GC tissues and paired adjacent normal tissues, respectively in cohort of this study (**a**), Lnc2Cancer 3.0 database (**b**) and GEPIA database (**c**). K–M Plotter Kaplan–Meier survival analysis of overall survival in GC patients based on HOXA11-AS expression (**d**) (*P* = 0.015). K–M Plotter Kaplan–Meier survival analysis of first progress survival in GC patients based on HOXA11-AS expression (**e**) (*P* = 0.084). ROC curve of HOXA11-AS diagnostic performance in gastric cancer patients (**f**). Association between the expressions of HOXA11-AS with that of miR-124-3p (**g**) and ITGB3 (**h**) in GC tissues. Relative luciferase activity of different transfection group in dual luciferase reporter assay (**i**). * *P* < 0.01

To investigate the function of lncRNA HOXA11-AS in gastric cancer, we first assessed the expression levels of HOXA11-AS in gastric cancer tissues and adjacent normal tissues of patients. The results showed that lncRNA HOXA11-AS expression of GC tissues was statistically higher than that of adjacent normal tissues (*P* < 0.001, Fig. 1a). Besides, we detected that HOXA11-AS expression of three human GC cell lines was higher than that of normal gastric mucosa cell line (see Additional file 2: Figure S1a). According to the results of Lnc2Cancer 3.0 (*P* < 0.001, Fig. 1b) and GEPIA database (*P* < 0.01, Fig. 1c), HOXA11-AS expression of stomach adenocarcinoma (STAD) tissues was also higher than that of normal stomach tissues. Besides, GEO datasets, GSE103236, GSE158662 and GSE79973, were used to validate the expression difference (see Additional file 1: Table S3). And HOXA11-AS expression of our GC patients’ cohort was statistically associated with clinical characteristics of GC patients, including tumor size, T stage, lymph node metastasis and patients’ clinical stage (see Additional file 3: Figure S2a–f). Moreover, the results from Lnc2Cancer 3.0 database also indicated that HOXA11-AS expression was correlated with clinical stage, which was consistent with our results (see Additional file 3: Figure S2g). Results of both our cohort and Lnc2Cancer 3.0 cohort showed that HOXA11-AS expression increased with the tumor progression during I to III stage of gastric cancer but decreased in IV stage patients (see Additional file 3: Figure S2 a and g), and the same is observed in tumor T stage of our cohort (see Additional file 3: Figure S2 b). Besides, survival data of GC patients in our study was not able to be tracked. However, survival analysis based on Kaplan–Meier Plotter (K–M Plotter) database showed that overall survival (OS) and first-progression (FP) survival of GC patients with low expression of HOXA11-AS were better than that of patients with high expression of HOXA11-AS (OS, *P* = 0.015, Fig. 1d; FP, *P* = 0.084, Fig. 1e). In this analysis, best cutoff to split patients based on survival was selected as 3 and all GC patients in spite of TNM stage and differentiation degrees were included. We next assessed the diagnostic performance of HOXA11-AS expression in gastric cancer. Comparison of cancerous and non-cancerous gastric tissues using ROC analysis indicated the AUC of HOXA11-AS expression was 0.86, indicating moderate to high diagnostic performance (Fig. 1f). In addition, we also performed expression correlation analysis between HOXA11-AS and miR-124-3p as well as ITGB3. HOXA11-AS expression was negatively correlated with miR-124-3p expression and positively correlated with ITGB3 expression (Fig. 1g, h). Luciferase reporter assay showed miR-124-3p mimics significantly inhibited the luciferase activity in HEK-293 T cells transfected with psiCEHCK-2-HOXA11-11-WT, but not in HEK-293 T cells transfected with psiCEHCK-2-HOXA11-11-Mut (Fig. 1i). According to starBase V2.0 [17] and lncRNASNP2 [18] databases, the binding signature between miR-124-3p and lncRNA HOXA11-AS was shown in Additional file 4: Figure S3.

### HOXA11-AS acts as a ceRNA to sponge miR-124-3p and up-regulate ITGB3 expression in vitro

**Fig. 2:**
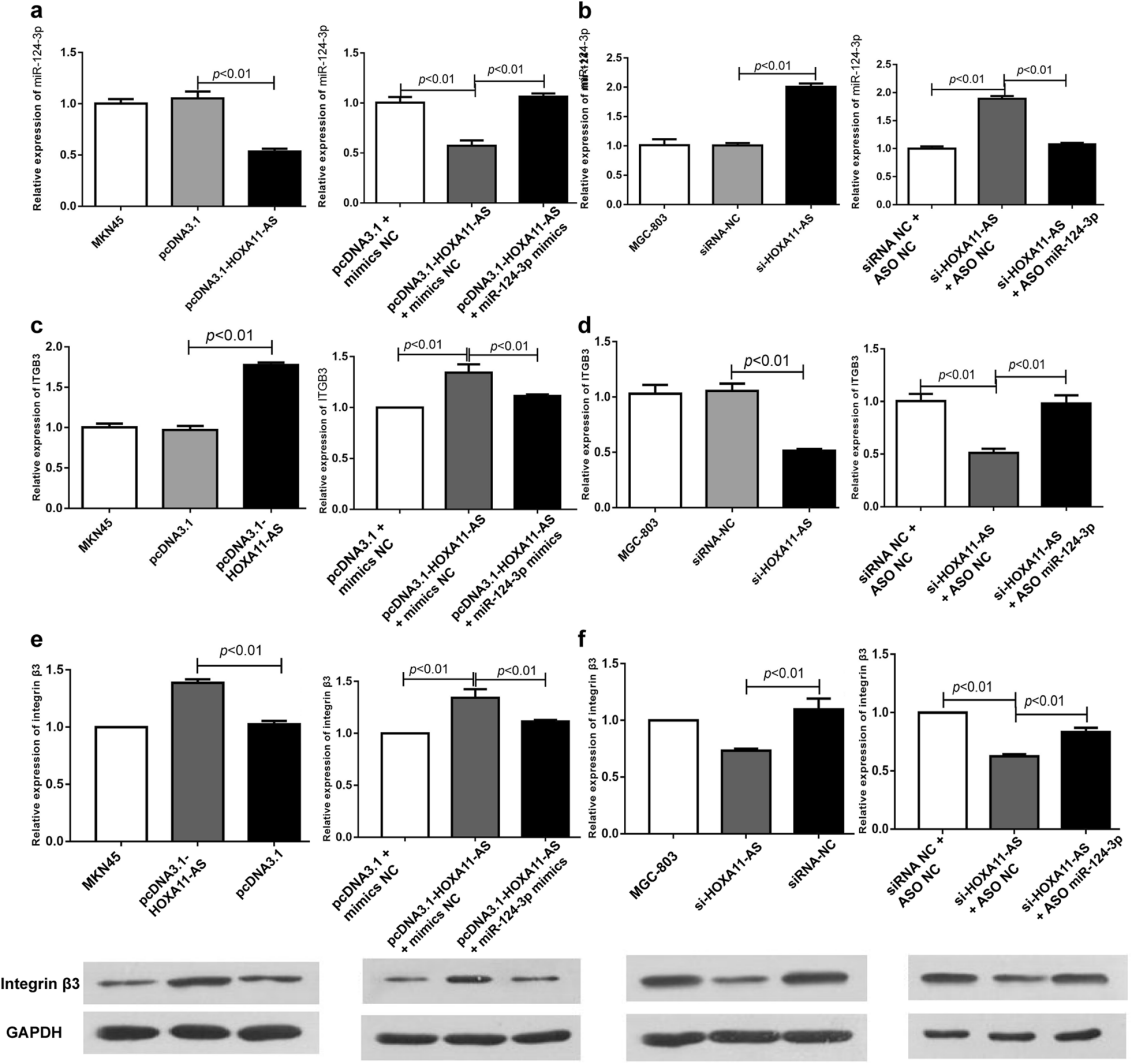
HOXA11-AS acts as a ceRNA to sponge miR-124-3p and up-regulate ITGB3 expression in vitro. **a** Overexpression of HOXA11-AS down-regulated the expression of miR-124-3p and miR-124-3p mimics partially reversed the effects. **b** Silence of HOXA11-AS up-regulated the expression of miR-124-3p and miR-124-3p inhibitors partially reversed the effects. **c**, **e** Overexpression of HOXA11-AS up-regulated the mRNA (**c**) and protein (**e**) expression of ITGB3 and miR-124-3p mimics partially reversed the effects. **d**, **f** Silence of HOXA11-AS down-regulated the mRNA (**c**) and protein (**e**) expression of ITGB3 and miR-124-3p inhibitors partially reversed the effects

In order to further explore the relation between lncRNA HOXA11-AS and miR-124-3p as well as ITGB3, we conducted lncRNA HOXA11-AS overexpression experiments, silencing experiments and rescue experiments in gastric cancer cell lines. Firstly, we detected the basal levels of HOXA11-AS and ITGB3 in normal gastric mucosal epithelial cells GES-1, as well as gastric cancer cells MNK45, AGS and MGC803 (see Additional file 2: Figure S1). Generally, the expression levels of HOXA11-AS and ITGB3 in GC cells were higher than that in normal gastric cells. And among three GC cell lines, MNK45 cells showed the lowest expression of HOXA11-AS and ITGB3, while MGC803 cells showed the highest. According to the results, we selected MNK45 cells for overexpression experiments, and MGC803 cells for silencing experiments. We found that miR-124-3p expression was significantly inhibited when HOXA11-AS was overexpressed in MKN45 cells (Fig. 2a left). By transfecting miR-124-3p mimics to these cells, miR-124-3p expression was restored (Fig. 2a right). While knockdown of HOXA11-AS markedly increased the miR-124-3p expression (Fig. 2b left). And miR-124-3p inhibitor could partially reverse above effects (Fig. 2b right). For transcriptional expression of ITGB3, we designed the similar experiments to evaluate its expression levels by using RT-PCR. HOXA11-AS overexpression increased the expression of ITGB3 and the addition of miR-124-3p mimics partially reversed the increase of ITGB3 (Fig. 2c). While HOXA11-AS knockdown inhibited the expression of ITGB3 and miR-124-3p inhibitor partially reversed the decrease of ITGB3 (Fig. 2d). Notably, the change trend of ITGB3 protein level was the same (Fig. 2e, f). To sum up, it was demonstrated that the expression level of HOXA11-AS was negatively correlated with that of miR-124-3p and was positively correlated with that of ITGB3.

### HOXA11-AS promotes the migration, invasion and proliferation of gastric cancer cells in vitro

**Fig. 3:**
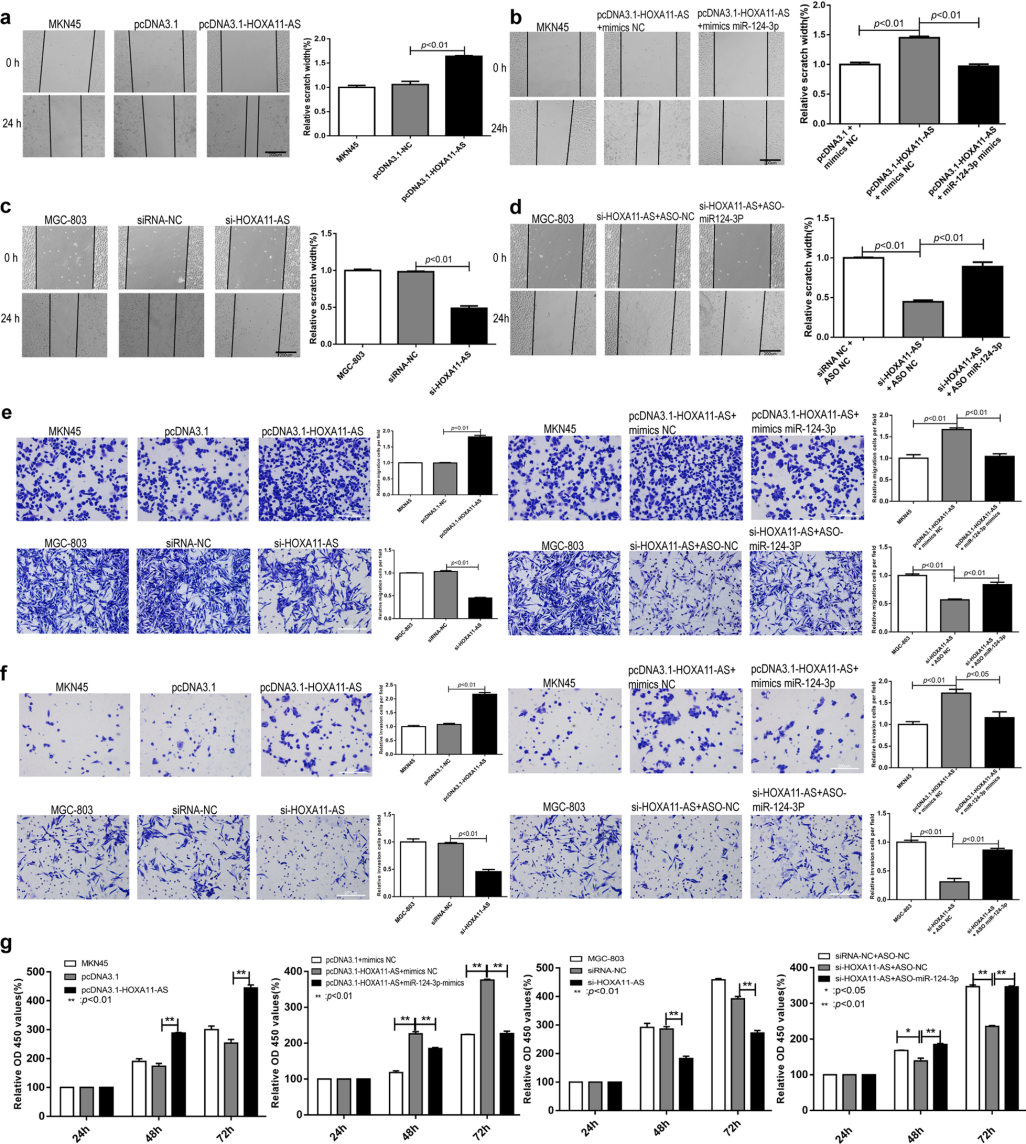
HOXA11-AS changes the migration, invasion and proliferation of gastric cancer cells in vitro. **a**, **b** the results of wound-healing assay through HOXA11-AS overexpression (**a**) and miR-124-3p mimics (**b**). **c**, **d** the results of wound-healing assay through HOXA11-AS silence (**c**) and miR-124-3p inhibitors (**d**). **e** The results of trans-well migration assay through HOXA11-AS overexpression, HOXA11-AS silence, miR-124-3p mimics and miR-124-3p inhibitors. **f** The results of trans-well invasion assay through HOXA11-AS overexpression, HOXA11-AS silence, miR-124-3p mimics and miR-124-3p inhibitors. **g** The results of CCK-8 cell proliferation assay through HOXA11-AS overexpression, miR-124-3p mimics, HOXA11-AS silence and miR-124-3p inhibitors. * *P* < 0.05, ** *P* < 0.01, *** *P* < 0.001

Functionally, we conducted wound-healing assays to determine the relative migration distance of cells;Trans-well assays to detect the migration and invasion ability of tumor cells; and CCK-8 assays to assess cell proliferation. Through wound-healing assay, we found that HOXA11-AS overexpression increased the relative migration distance of MKN45 gastric cells (*P* < 0.01, Fig. 3a) and the co-transfection with miR-124-3p mimics partially reversed the increase of migration ability (*P* < 0.01, Fig. 3b). On the contrary, silence of HOXA11-AS in MGC-803 gastric cancer cells decreased cells relative migration distance (*P* < 0.01, Fig. 3c) and the miR-124-3p inhibitor slightly reversed the migration ability of MGC-803 cells (*P* < 0.01, Fig. 3d). We observed similar results through trans-well migration and invasion assay that HOXA11-AS overexpression increased the migration and invasion ability of GC cells and silence of HOXA11-AS decreased malignant potential of GC cells (*P* < 0.01, Fig. 3e, f). And the miR-124-3p mimics or inhibitors could partially restore the changes of migration and invasion ability caused by overexpression or silence of HOXA11-AS, respectively (Fig. 3e, f). CCK-8 cell proliferation assay showed that proliferation ability of MKN45 after transfection of pcDNA3.1-HOXA11-AS was remarkably increased in comparison with that of MKN45 cells transfected with pcDNA3.1-control (*P* < 0.01, Fig. 3g). And the increase of cells proliferation was partially reversed by miR-124-3p mimics (*P* < 0.01, Fig. 3g). Besides, silence of HOXA11-AS in MGC-803 inhibited cells proliferation and the miR-124-3p inhibitor partially reversed the decrease of proliferation ability (*P* < 0.01, Fig. 3g). To sum up, it was demonstrated that HOXA11-AS could promote the migration, invasion and proliferation of gastric cancer cells in vitro.

### HOXA11-AS up-regulates ITGB3 expression and promotes tumor growth by sponging miR-124-3p in animal models

**Fig. 4:**
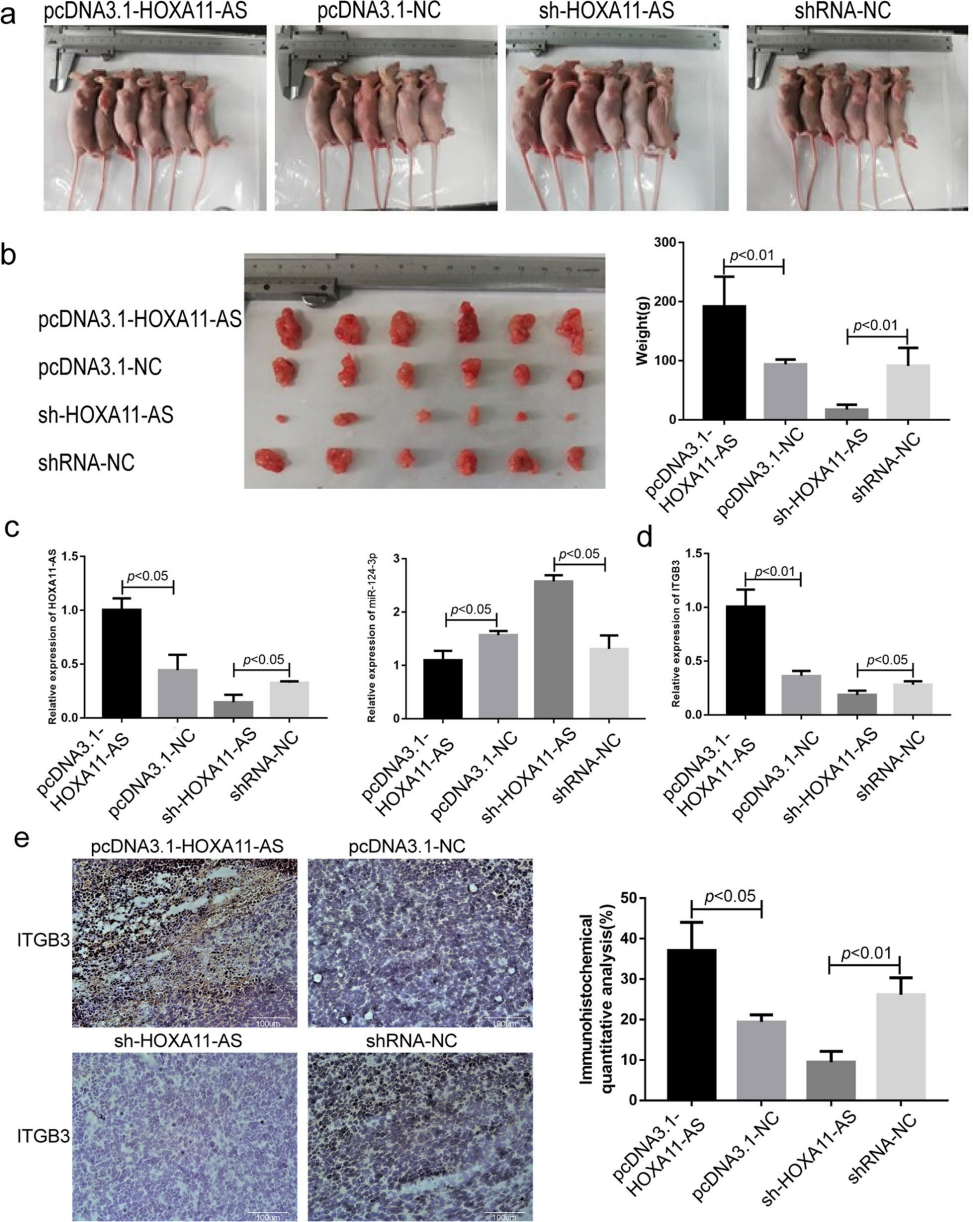
HOXA11-AS up-regulates ITGB3 expression and promotes tumor growth by sponging miR-124-3p in animal models. **a**, **b** The tumor size and weight of mice in pcDNA3.1-HOXA11-AS, pcDNA3.1-negative control (NC), sh-HOXA11-AS and sh-NC group. **c** The expression of HOXA11-AS (left) and miR-124-3p (right) in pcDNA3.1-HOXA11-AS, pcDNA3.1- NC, sh-HOXA11-AS and sh-NC group. **d**, **e** The mRNA (**d**) and protein (**e**) expression of ITGB3 in pcDNA3.1-HOXA11-AS, pcDNA3.1- NC, sh-HOXA11-AS and sh-NC group

We further evaluated the function of HOXA11-AS on tumor growth in vivo using xenograft gastric cancer mouse model. AGS gastric cancer cells were transfected with pcDNA3.1-negative control (NC), pcDNA3.1-HOXA11-AS, sh-NC or sh-HOXA11-AS and implanted into the subcutaneous tissue of nude mice back. Tumor histopathological H&E staining results were shown in Additional file 5: Figure S4. The tumor size of mice in HOXA11-AS overexpression group was statistically larger than that in control group (Fig. 4a, b). And the tumor size of mice in HOXA11-AS silence group was statistically smaller than that in control group (Fig. 4a, b). And the miR-124-3p expression was remarkably down-regulated in pcDNA3.1-HOXA11-AS mice and up-regulated in sh-HOXA11-AS mice (*P* < 0.05, Fig. 4c). Simultaneously, the mRNA and protein expression of ITGB3 in tumor tissues increased in HOXA11-AS overexpression mice and decreased in HOXA11-AS silence mice (*P* < 0.05, Fig. 4d and e). Tumor Ki67 and CD34 IHC staining results were shown in Additional file 5: Figure S4.

## Discussion

**Fig. 5:**
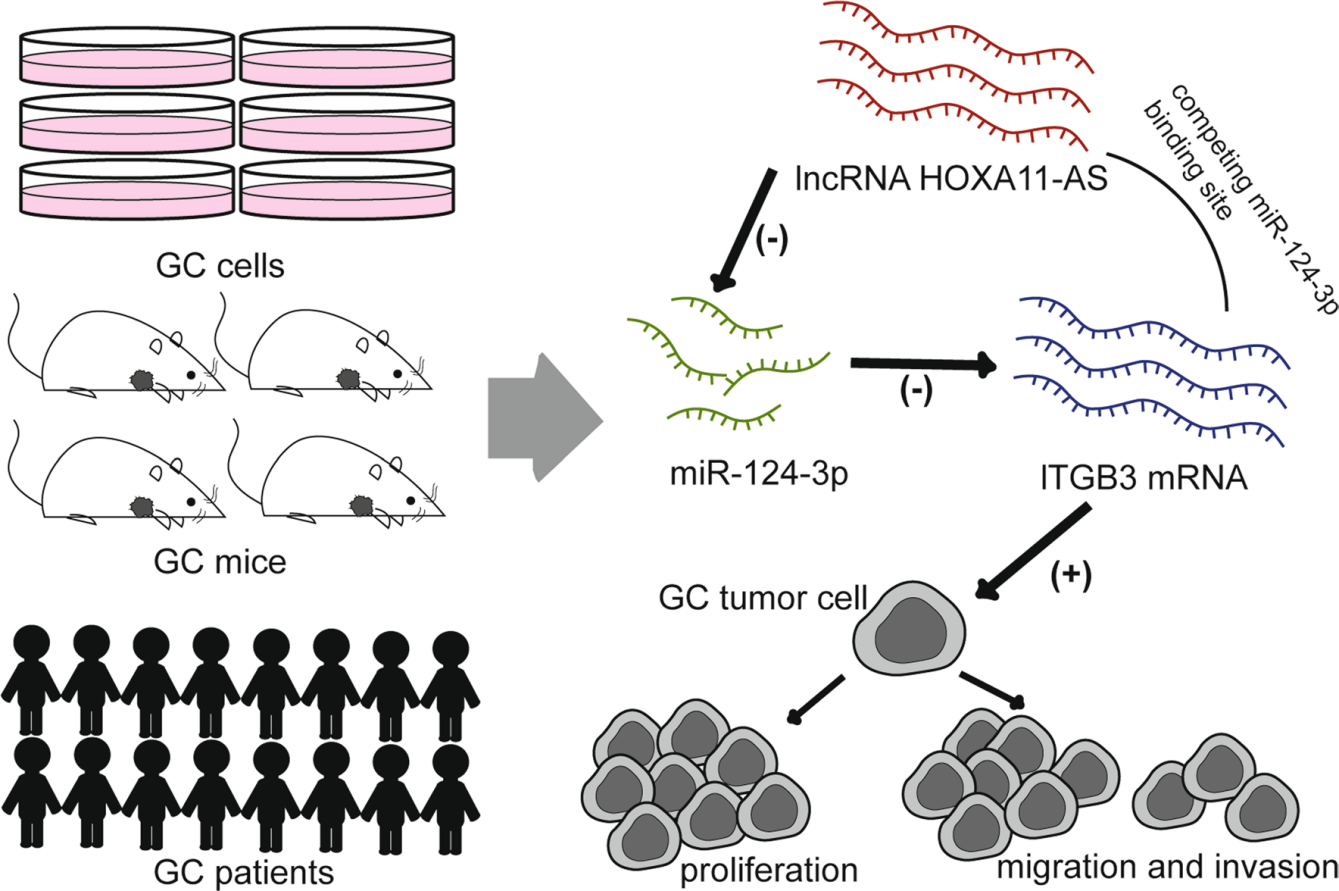
HOXA11-AS could promote progression and metastasis of GC by sponging miR-124-3p to up-regulate ITGB3 expression. The experiments in patients, cells and animals level demonstrated that HOXA11-AS can competitively bind to miR-124-3p with ITGB3 so that down-regulate miR-124-3p and up-regulate ITGB3 expression to promote the proliferation, migration and invasion of gastric cancer

GC carcinogenesis, progression and metastasis are complex pathological process, which involves the abnormality of complicated molecular regulatory network. Till now, the underlying regulating details of DNAs, RNAs and proteins in GC still remain unclear. In our previous study, single nucleotide polymorphisms (SNPs) in the microRNA-binding sites of ITGB3 mRNA 3′ UTR region were associated with susceptibility and stage of GC [19]. In our further study, we reported that miR-124-3p can inhibit the migration and invasion of GC by inhibiting ITGB3 expression [7]. As a result, we identified miR-124-3p/ITGB3 pathway as one of important mechanisms in the proliferation and invasion of GC. Subsequently in this study, we further demonstrated that HOXA11-AS could promote progression and metastasis of GC by sponging miR-124-3p to up-regulate ITGB3 expression (Fig. 5). By binding with miR-124-3p, ITGB3 mRNA was down-regulated. HOXA11-AS can competitively bind to miR-124-3p with ITGB3 so that miR-124-3p was down-regulated and ITGB3 was up-regulated. Besides, we also demonstrated that HOXA11-AS expression of GC tissues was obviously higher than that of normal tissues; and that HOXA11-AS showed great diagnostic performance for GC. However, there are some seemingly mutually contradictory results in our study. For GC patients of different clinical stage, HOXA11-AS expression was also statistically different. HOXA11-AS expression was gradually increased in patients with stage I to III gastric cancer, while decreased in patients with stage IV gastric cancer. Survival analyses of K-M Plotter revealed that GC patients with high expression of HOXA11-AS showed shorter OS and FPS. But survival analyses from TCGA patients of Lnc2Cancer 3.0 database showed contradictory results that STAD patients with high expression of HOXA11-AS showed better survival. These seemingly contradictory conclusions may cause by several reasons. Firstly, the results of our cohort and KM-plotter cohort are same that patients with higher HOXA11-AS expression have better outcomes, which was also consistent with our results of mechanism study. However, the result of Lnc2Cancer 3.0 cohort was opposite. The patient composition of above two cohorts was not identical. Race, ethnicity, gender, age, the tumor size, clinical stage, pathologic grade and et al. of patients in the cohorts are different. And these factors are correlated with HOXA11-AS expression. In addition, it is possible that HOXA11-AS only participates in triggering tumor progression and metastasis, and does not play a major role in the maintenance of advanced gastric cancer after metastasis. Hence, the roles of HOXA11-AS in survival and prognosis of GC patients still need more investigation. In fact, the evidences for differential expression of HOXA11-AS in GC versus normal tissues and diagnostic performance of HOXA11-AS are strong in multiple GC patients’ cohorts. Consequently, we suggested that HOXA11-AS may reasonably serve as a promising early diagnostic biomarker and a potential therapeutic target for the early prevention from metastasis of GC.

In addition to our studies, several studies also reported the important roles of HOXA11-AS in GC. For instance, Liu et al. also reported that HOXA11-AS expression of GC tissues was higher compared with that of normal tissues [20]. Furtherly, Sun et al. revealed that higher lncRNA HOXA11-AS expression was closely related with worse progression-free survival (PFS) and OS rate. And HOXA11-AS expression was positively correlated with tumor size, differentiation degree and clinical pathological stage [21]. Besides, other studies also reported that HOXA11-AS can promote cell proliferation, invasion and metastasis of GC through modulating different pathways [20, 22, 23]. Another study by Xu et al. observed that the suppression of HOXA11-AS can alleviate the postoperative distant recurrence of GC [24]. In conclusion HOXA11-AS can promote GC migration and invasion. However, other molecular pathways may also exist in GC and play crucial actions in the invasion and migration of GC. The significant value of HOXA11-AS is that it can be concerned as a potential diagnostic marker and therapeutic target for GC.

Furthermore, the roles of HOXA11-AS in other tumors have been explored in several studies, although the conclusions are inconsistent. Zhang et al. reported that HOXA11-AS was higher in non-small cell lung cancer (NSCLC) tissues than that in normal tissues [25]. Wang et al. proposed HOXA11-AS could be an independent prognostic factor for glioblastoma patients [26]. Another study by Xu et al. drew the consistent conclusion in glioma cells that lncRNA HOXA11-AS can sponge miR-214-3p to promote EZH2 expression and act as an oncogenic factor [27]. Besides, miR-124-3p was defined as another molecular targeted by HOXA11-AS to promote malignant progression of glioma [28]. In osteosarcoma, hepatocellular carcinoma and colorectal cancer (CRC) studies, HOXA11-AS has been identified as a ceRNA to regulate gene expression by sponging specific miRNA to promote tumor progression [8, 29–31]. In the fracture healing studies, lncRNA HOXA11-AS can inhibit osteoblast proliferation and promote osteoblast apoptosis [32]. However, some studies drew the totally opposite conclusion that HOXA11-AS have tumor suppressor function. For instance, Li et al. found that HOXA11-AS expression of CRC tissues and cell lines was lower than that of normal tissues and cell lines; and that the low expression of HOXA11-AS was significantly associated with the advanced tumor stage and the poorer prognosis [33]. Another study in ovarian cancer revealed that HOXA11-AS showed lower expression in ovarian cancer tissues and played a tumor suppressor functions [34]. In conclusion, HOXA11-AS shows tissue-specific expression patterns and plays oncogene or tumor-suppressor functions according to different circumstance manners (tumor types and regulation of gene expression). However, in GC, the conclusions of the current study are uncontroversial.

Based on our previous study, this study revealed that HOXA11-AS promoted the progression and invasion of GC through modulating miR-124-3p/ITGB3 pathway. Through validation experiments in vitro and in vivo, we proved one of crucial mechanisms of HOXA11-AS in GC progression and metastasis, yet other regulating networks of HOXA11-AS underlying the complex physiological process remain unclear. For instance, Sun et al. proposed HOXA11-AS mechanism model in which the EZH2/HOXA11-AS/LSD1 complex and HOXA11-AS/miR-1297/EZH2 cross-talk and function as critical effectors in GC carcinogenesis and progression [21]. According to the results of Liu’s study, HOXA11-AS could interact with WDR5, EZH2 and STAU1; and promoteβ-catenin expression, repress P21 and KLF2 expression to promote GC migration and metastasis [20]. Guo et al. revealed that HOXA11-AS can promote migration and invasion of GC through modulating miR-148a/WNT1/β-catenin pathway [22]. A preliminary mechanism study by database analysis and western blot analysis showed SRSF1 may be the target of HOXA11-AS in GC [23]. In addition, effective mechanisms of HOXA11-AS in other tumors were far more complicated, such as miR-149-3p/epithelial mesenchymal transition (EMT) pathway in CRC [29] and miR-a24-3p/ROCK1 in osteosarcoma [8]. In fact, cancer initiation and progression are indeed the complex biological processes concerning the activating or inhibiting of a series of molecules and pathways. It’s well-known that malignancies always show the significant individual differences and intra-tumor heterogeneity, in other words, no two tumors are exactly the same in the world. Furthermore, lncRNAs play various actions by many different mechanisms (in Cis or in Trans) and participate in almost all steps of tumor development and metastasis [35]. The complicated pathways underlying tumor phenotype are unknown to human beings. Consequently, effective mechanisms of HOXA11-AS may be tissue-specific and even different from individuals, which need more explorations. Our study will contribute to the advancement of precision medicine and tumor personalized diagnosis and treatment, although the future long way to go.

In addition, several studies explored the regulation mechanisms of HOXA11-AS. Xu et al. revealed in GC that lncRNA PTCSC3 can inhibit the expression of HOXA11-AS and functions as a tumor suppressor [24]. And Sun et al. found E2F1 may be involved in HOXA11-AS activation in gastric cancer cells [21]. Besides, CTCF (CCCTC-binding factor) was validated to activate HOXA11-AS transcription in prostate cancer cells and consequently to facilitate cell proliferation and migration by targeting miR-518b/ACTN4 [36]. Currently, regulation mechanisms of lncRNA HOXA11-AS expression are merely clear. Therefore, this will be our future research direction.

Collectively, results in reported studies and our experiments indicate that lncRNA HOXA11-AS is a crucial factor in the regulation of tumor genesis and progression. In GC, both our study and reported literatures showed the significant high expression of HOXA11-AS. And its diagnostic performance was confirmed in our patients cohort and the prognostic performance was tested in database cohort. Certainly, the diagnostic and prognostic potentials should be validated furtherly in multicenter, prospective, large-sample cohorts of GC patients. Actually, studies on the mechanisms of HOXA11-AS in GC and other malignancies have hinted prosperous future that HOXA11-AS serve as a quite promising biomarker in clinical diagnosis and basic research.

## Conclusions

In conclusion, our collective findings demonstrated that the lncRNA HOXA11-AS can activate ITGB3 expression to promote the migration and invasion of gastric cancer by sponging miR-124-3p. We suggested that HOXA11-AS may reasonably serve as a promising diagnostic biomarker and a potential therapeutic target of gastric cancer.

## Supplementary Information

**Additional file 1: Table S1.** the primer sequence of the wild-type and mutant HOXA11-AS. **Table S2.** the primer sequence for RT-PCR. **Table S3.** HOXA11-AS expression in gastric normal vs. cancer tissues ofGEO datasets.

**Additional file 2: Figure S1.** Basal levels of HOXA11-AS and ITGB3 expression in GES-1, MKN45, AGS andMGC803 cells. (a) HOXA11-AS expression in GES-1, MKN45, AGS and MGC803 cells. (b)ITGB3 RNA expression in GES-1, MKN45, AGS and MGC803 cells. (c) Integrin β3 proteinexpression in GES-1, MKN45, AGS and MGC803 cells.

**Additional file 3: Figure S2.** Association between the expression of HOXA11-ASand clinical characteristics of GC patients. (a) HOXA11-AS expression with clinicaltumor stage I to IV (one-way ANOVA). (b) HOXA11-AS expression with tumor Tstage of TNM-staging system (one-way ANOVA). (c) HOXA11-AS expression with Nstage of TNM-staging system (one-way ANOVA). (d) HOXA11-AS expression with Mstage of TNM-staging system (t test). (e) HOXA11-AS expression with grade ofdifferentiation (t test). (f) HOXA11-AS expression with tumor size (≥5cm and < 5cm) (t test). HOXA11-AS expression of STAD patients in different clinical stage (g)(Y-axis was log2 (TPM + 1) representing gene expression levels; X-axis wastumor stage; and results were from Lnc2Cancer 3.0 database). Kaplan–Meiersurvival curve of STAD patients according to lncRNA HOXA11-AS expression (h) (*P* value was accessed by using log-ranktest; and results were from Lnc2Cancer 3.0 database).

**Additional file 4: Figure S3.** The binding signature between miR-124-3p and lncRNA HOXA11-AS sequenceaccording to StarBase V2.0 and lncRNASNP2 databases.

**Additional file 5: Figure S4.** Histological characteristics of mice tumor in pcDNA3.1-HOXA11-AS,pcDNA3.1- NC, sh-HOXA11-AS and sh-NC group. (a) Tumor histopathological H&Estaining results. (b and c) Tumor Ki67 (b) and CD34 (c) IHC staining results.

## Data Availability

All data generated in this study are included in the main text and additional materials.

## Abbreviations

HOXA11-AS: Homeobox A11 antisense RNA
GC: Gastric cancer
EZH2: Enhancer of zeste homolog 2
LSD1: Lysine-specific histone demethylase 1
WDR5: WD repeat domain 5
STAU1: Staufen double-stranded RNA binding protein 1
P21 (CDKN1A): Cyclin-dependent kinase inhibitor 1A
KLF2: Kruppel Like Factor 2
SRSF1: Serine and Arginine Rich Splicing Factor 1
ROCK1: Rho Associated Coiled-Coil Containing Protein Kinase 1
PTCSC3: Papillary thyroid carcinoma susceptibility candidate 3
E2F1: E2F transcription factor 1
ACTN4: Actinin alpha 4
