# Supplementary material for: The long non-coding RNA HOXA11-AS activates ITGB3 expression to promote the migration and invasion of gastric cancer by sponging miR-124-3p
Source: Cancer Cell Int. 2021 Oct 29;21:576. doi: 10.1186/s12935-021-02255-6 (PMC8556882; doi:10.1186/s12935-021-02255-6)
Supplement: Supplementary file 1 — Additional file 1: Table S1. the primer sequence of the wild-type and mutant HOXA11-AS. Table S2. the primer sequence for RT-PCR. Table S3. HOXA11-AS expression in gastric normal vs. cancer tissues ofGEO datasets. [file 12935_2021_2255_MOESM1_ESM.docx]

Table S1 the primer sequence of the wild-type and mutant HOXA11-AS.

| The primer name | The primer sequence |
| --- | --- |
| HOXA11-AS-MUT-F | TCGAGCCGAAGCGCTTTACACGGAACGTTT |
| HOXA11-AS-MUT-R | AAACGTTCCGTGTAAAGCGCTTCGGC |
| HOXA11-AS-WT-F | TCGAGCCGAAGCGCTTTAGTGCCTTCGTTT |
| HOXA11-AS-WT-R | AAACGAAGGCACTAAAGCGCTTCGGC |

Table S2 the primer sequence for RT-PCR.

| The primer name | The primer sequence |
| --- | --- |
| GAPDH-F | 5’-ACACCCACTCCTCCACCTTT-3’ |
| GAPDH-R | 5’-TTACTCCTTGGAGGCCATGT-3’ |
| HOXA11-AS-F | 5’-GAGTGTTGGCCTGTCCTCAA-3’ |
| HOXA11-AS-R | 5’-TTGTGCCCAGTTGCCTGTAT-3’ |
| ITGB3-F | 5’-GTGACCTGAAGGAGAATCTGC-3’ |
| ITGB3-R | 5’-CCGGAGTGCAATCCTCTGG-3’ |
| miR-124-3p-F | 5'-GCTTAAGGCACGCGG-3' |
| miR-124-3p-R | 5'-GTGCAGGGTCCGAGG-3' |
| miR-124-3p-RT | 5'-GTCGTATCCAGTGCAGGGTCCGAGGTATTCGCACTGGATA CGACGGCATTC-3' |
| U6-F | 5'-CTCGCTTCGGCAGCACATATACT-3' |
| U6-R | 5'-ACGCTTCACGAATTTGCGTGT-3' |
| U6-RT | 5'-AAAATATGGAACGCTTCACGAATT-3' |

Table S3 HOXA11-AS expression in gastric normal vs. cancer tissues of GEO datasets.

| Gene name | GEO datasets | | Experiment name | Comparison | log_2 fold change | P-value |
| --- | --- | --- | --- | --- | --- | --- |
| HOXA11-AS | GSE103236 | Microarray gene expression analysis for early and advanced gastric adenocarcinoma vs normal adjacent tissue | | Gastric normal tissues vs. gastric cancer | -2.72266149 | 4.57e-03 |
| HOXA11-AS | GSE158662 | mRNA expression of gastric cancer and paracancerous tissues | | Paracancerous vs. gastric cancer | -1.19129574 | 2.31e-02 |
| HOXA11-AS | GSE79973 | Expression data from gastric cancer and paired normal tissues | | Gastric mucosa vs. gastric adenocarcinoma | -1.1519393 | 1.29e-01 |
