# Supplementary figures and images for: The long non-coding RNA HOXA11-AS activates ITGB3 expression to promote the migration and invasion of gastric cancer by sponging miR-124-3p
Source: Cancer Cell Int. 2021 Oct 29;21:576. doi: 10.1186/s12935-021-02255-6 (PMC8556882; doi:10.1186/s12935-021-02255-6)

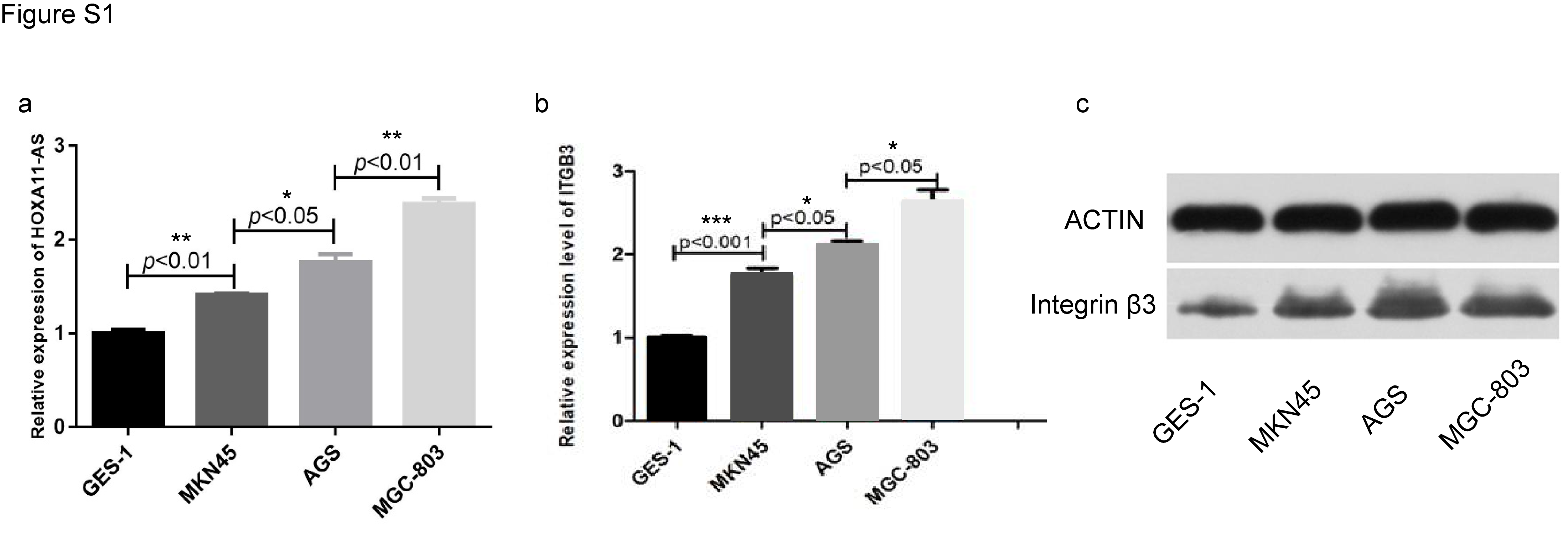

Supplement: Supplementary file 2 — Additional file 2: Figure S1. Basal levels of HOXA11-AS and ITGB3 expression in GES-1, MKN45, AGS andMGC803 cells. (a) HOXA11-AS expression in GES-1, MKN45, AGS and MGC803 cells. (b)ITGB3 RNA expression in GES-1, MKN45, AGS and MGC803 cells. (c) Integrin β3 proteinexpression in GES-1, MKN45, AGS and MGC803 cells. [file 12935_2021_2255_MOESM2_ESM.tif]

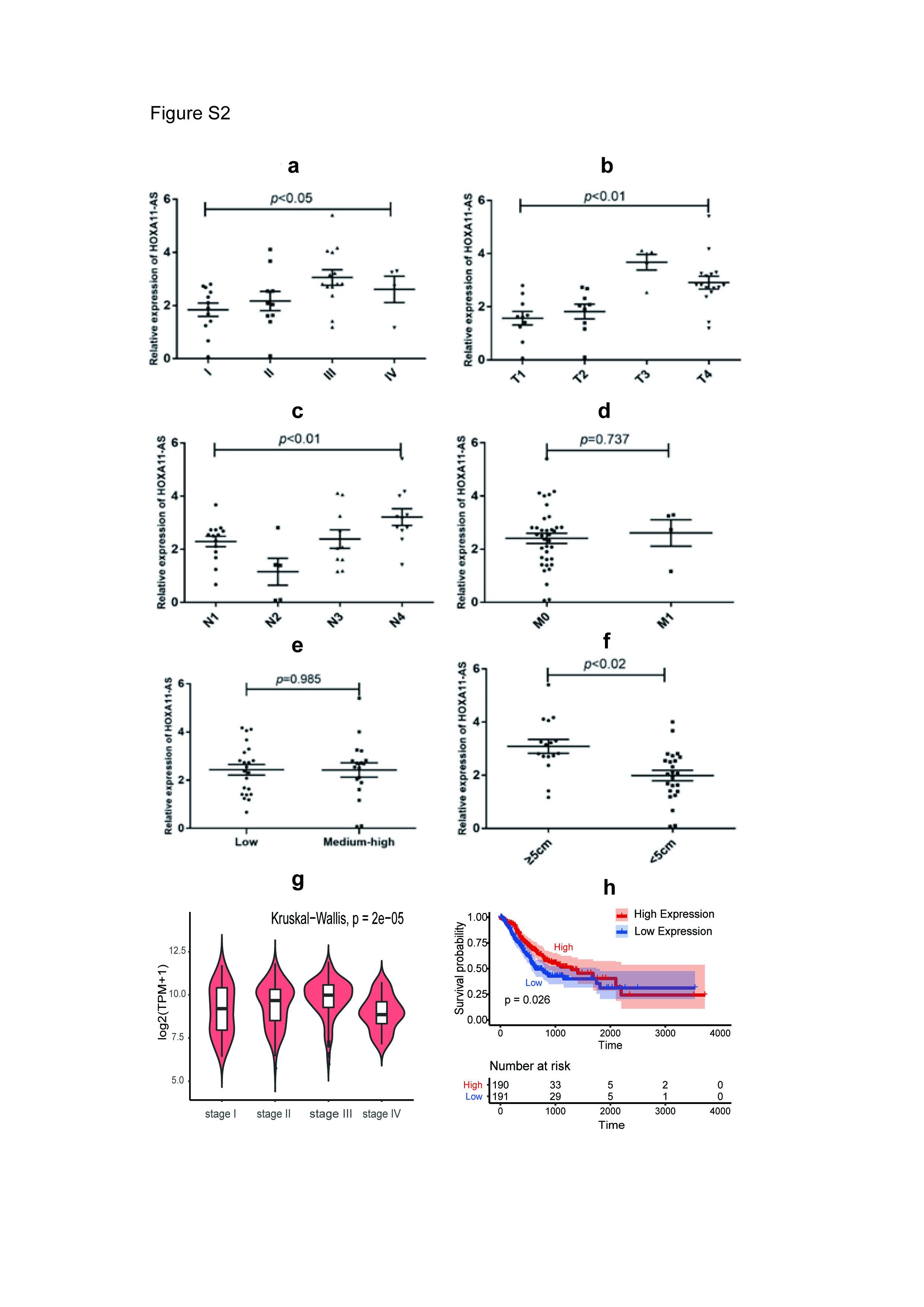

Supplement: Supplementary file 3 — Additional file 3: Figure S2. Association between the expression of HOXA11-ASand clinical characteristics of GC patients. (a) HOXA11-AS expression with clinicaltumor stage I to IV (one-way ANOVA). (b) HOXA11-AS expression with tumor Tstage of TNM-staging system (one-way ANOVA). (c) HOXA11-AS expression with Nstage of TNM-staging system (one-way ANOVA). (d) HOXA11-AS expression with Mstage of TNM-staging system (t test). (e) HOXA11-AS expression with grade ofdifferentiation (t test). (f) HOXA11-AS expression with tumor size (≥5cm and < 5cm) (t test). HOXA11-AS expression of STAD patients in different clinical stage (g)(Y-axis was log2 (TPM + 1) representing gene expression levels; X-axis wastumor stage; and results were from Lnc2Cancer 3.0 database). Kaplan–Meiersurvival curve of STAD patients according to lncRNA HOXA11-AS expression (h) (P value was accessed by using log-ranktest; and results were from Lnc2Cancer 3.0 database). [file 12935_2021_2255_MOESM3_ESM.tif]

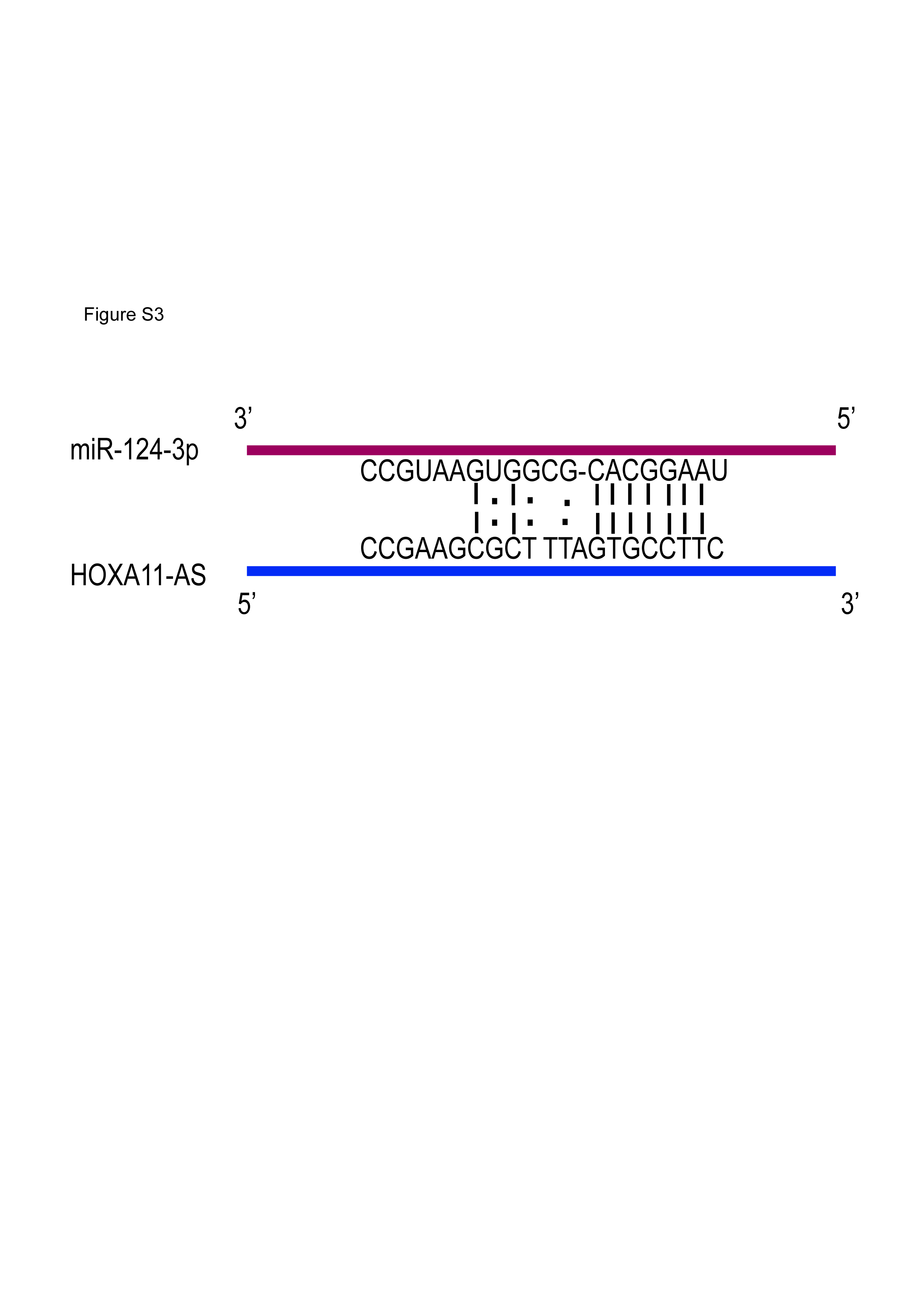

Supplement: Supplementary file 4 — Additional file 4: Figure S3. The binding signature between miR-124-3p and lncRNA HOXA11-AS sequenceaccording to StarBase V2.0 and lncRNASNP2 databases. [file 12935_2021_2255_MOESM4_ESM.tif]

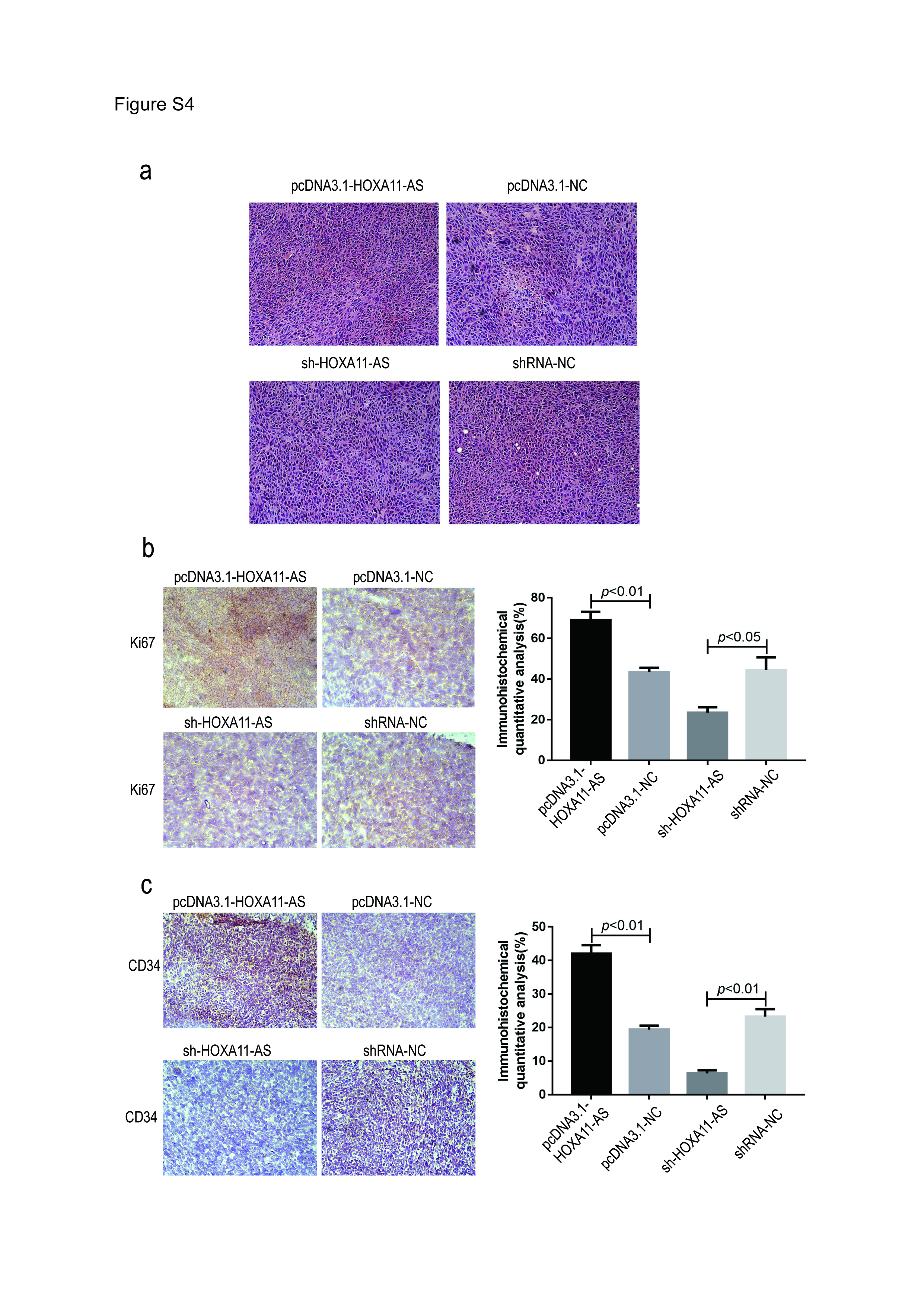

Supplement: Supplementary file 5 — Additional file 5: Figure S4. Histological characteristics of mice tumor in pcDNA3.1-HOXA11-AS,pcDNA3.1- NC, sh-HOXA11-AS and sh-NC group. (a) Tumor histopathological H&Estaining results. (b and c) Tumor Ki67 (b) and CD34 (c) IHC staining results. [file 12935_2021_2255_MOESM5_ESM.tif]
